# Carbon-ion radiotherapy for lymph node oligo-recurrence: a multi-institutional study by the Japan Carbon-Ion Radiation Oncology Study Group (J-CROS)

**DOI:** 10.1007/s10147-019-01440-y

**Published:** 2019-04-09

**Authors:** Noriyuki Okonogi, Takuya Kaminuma, Tomoaki Okimoto, Makoto Shinoto, Naoyoshi Yamamoto, Shigeru Yamada, Kazutoshi Murata, Tatsuya Ohno, Yoshiyuki Shioyama, Hiroshi Tsuji, Takashi Nakano, Tadashi Kamada

**Affiliations:** 1 QST Hospital, National Institutes for Quantum and Radiological Science and Technology, 4-9-1 Anagawa, Inage-ku, Chiba, 263-8555 Japan; 2 Department of Radiation Oncology, Gunma University Graduate School of Medicine, Maebashi, Gunma Japan; 3 Department of Radiology, Hyogo Ion Beam Medical Center, Tatsuno, Hyogo Japan; 4 Ion Beam Therapy Center, SAGA HIMAT Foundation, Saga, Japan

**Keywords:** Carbon-ion radiotherapy, Radiotherapy, Lymph nodes metastasis, Oligo-recurrence

## Abstract

**Background:**

The efficacy of carbon-ion radiotherapy (C-ion RT) for lymph node (LN) oligo-recurrence has only been evaluated in limited single-center studies. We aimed to investigate the benefit of C-ion RT for LN oligo-recurrence in a large multi-center study.

**Methods:**

Patients who received C-ion RT between December 1996 and December 2015 at 4 participating facilities and who met the following eligibility criteria were included: (i) histological or clinical diagnosis of LN recurrence; (ii) controlled primary lesion; (iii) no recurrence other than LN; (iv) LN recurrence involved in a single lymphatic site; and (v) age ≥ 20 years.

**Results:**

A total of 323 patients were enrolled. Median follow-up period was 34 months for surviving patients. The most common dose fractionation of C-ion RT was 48.0 Gy (relative biological effectiveness) in 12 fractions. Forty-seven patients had a history of RT at the recurrent site. The 2-year local control (LC) and overall survival (OS) rates after C-ion RT were 85% and 63%, respectively. Only 1 patient developed grade-3 toxicity. Factors such as LN diameter, histology, and history of previous RT did not correlate with LC. Smaller diameters (< 30 mm) and numbers (≤ 3) of LN metastases as well as longer disease-free intervals post-primary therapy (≥ 16 months) were associated with significantly better OS.

**Conclusions:**

C-ion RT for LN oligo-recurrence appeared to be effective and safe. C-ion RT may provide a survival benefit to patients with LN oligo-recurrence, particularly to those with few LN metastases, smaller LN diameters, and longer disease-free intervals.

**Electronic supplementary material:**

The online version of this article (10.1007/s10147-019-01440-y) contains supplementary material, which is available to authorized users.

## Introduction

Metastatic tumors have traditionally been managed with systemic treatment administered with palliative intent. The presence of metastases is considered a clinical manifestation of widespread microscopic disseminated disease. To that end, the concept of oligometastases was described in 1995 as a distinct clinical entity involving limited metastatic disease [1]; it was suggested that few metastases initially exist in patients with cancer of multiple types before malignant cells acquire widespread metastatic potential [1]. Regarding this point, Niibe et al. distinguished the states of oligometastases and oligo-recurrence [2]. Oligometastases is the state in which the patient shows distant relapse in only a limited number of regions. However, the concept of oligometastases includes the state of uncontrolled primary site with several distant metastases. Meanwhile, oligo-recurrence means the state of recurrence or metastases with a controlled primary lesion, meaning that all metastatic sites could be cured using local therapy [2]. Consistent with this concept, many studies have demonstrated improved survival and long-term disease control after surgical resection of metastases in cancer patients with certain histological subtypes [3–7].

Surgical resection of metastatic lesions is the preferred curative treatment. However, such surgeries are sometimes restricted in patients with oligo-recurrence because of technical obstacles, patient refusal, advanced age, or associated clinical comorbidities. In recent years, state of the art radiotherapy (RT) techniques, such as stereotactic body RT (SBRT), have shown clinical benefits for patients with oligo-recurrence while avoiding the risks associated with surgery [8, 9]. Growing evidence suggests that lung and liver metastases can be treated locally using SBRT, with low toxicity and excellent outcomes [10, 11]. However, the number of studies on the use of SBRT for lymph node (LN) metastasis is still limited.

Ion beams, such as protons and carbon ions (C-ions), provide a dose distribution that is superior to that of photons during cancer treatment. Additionally, C-ion beams are heavier than protons and provide a higher relative biological effectiveness (RBE) and thus a higher probability of tumor control, while delivering a smaller dose to the surrounding normal tissues [12, 13]. Several studies have shown the effectiveness of C-ion RT in treating LN oligo-recurrences of radioresistant tumors or for managing recurrences after other RT methods [14–16]. These studies suggest that C-ion RT for patients with LN oligo-recurrence could be a useful treatment option. However, available data have been derived from single institutions, were limited in the type of cancers included, and were extracted from a small number of patients. Hence, we conducted a retrospective multicenter study to investigate the efficacy of C-ion RT in patients with LN oligo-recurrence.

## Materials and methods

### Eligibility

This retrospective multicenter study was conducted within the framework of the Japan Carbon-Ion Radiation Oncology Study Group (J-CROS) and is registered with the University Hospital Medical Information Network Clinical Trials Registry (http://www.umin.ac.jp/ctr/index-j.htm), identification number UMIN000027300. The study was approved by the institutional review board of each participating institution and conducted in compliance with the Declaration of Helsinki.

Among patients who received C-ion RT between December 1996 and December 2015 at the 4 participating facilities (the Hospital of the National Institute of Radiological Sciences, Hyogo Ion Beam Medical Center, Gunma University Heavy Ion Medical Center, and the SAGA-HIMAT Foundation), those who met the following eligibility criteria were enrolled: (i) histological or clinical diagnosis of LN recurrence; (ii) controlled primary tumor; (iii) no recurrence other than that of the solitary LN; (iv) LN recurrence involved at a single lymphatic site; and (v) 20 years of age or older. Based on these criteria, 323 patients with LN oligo-recurrences from the 4 institutions were included in this study. An LN with a short-axis diameter of > 10 mm was defined as a metastatic LN in the present study.

### Treatment planning system and dose fractionation

Patients were positioned in customized cradles and immobilized with a low-temperature thermoplastic sheet. A set of 2.0–2.5-mm-thick computed tomography (CT) images were acquired for 3-dimensional treatment planning using the HiPLAN or Xio-N system. Patients received C-ion RT daily for 4 days/week (Tuesday through Friday). The radiation dose was calculated for the target volume and is expressed in Gy (RBE), which is defined as the physical dose multiplied by the RBE of the C-ions [17]. Treatment consisted of regional LN irradiation or prophylactic irradiation and local LN boost; the clinical target volume included all swollen LNs and potentially microscopic disease. The planning target volume included the clinical target volume plus a 2–10 mm safety margin to account for intra- and inter-movement. The planning target volume was covered by ≥ 90% of the prescribed dose. A representative dose distribution of C-ion RT is shown in the supplementary figure. The dose fractionation schemes are listed in Table 1.

**Table 1:** Characteristics of carbon-ion radiotherapy

Dose fractionation schemes	Number of patients
36.0 Gy (RBE)/8 fr.	1
52.8 Gy (RBE)/8 fr.	1
60.0 Gy (RBE)/8 fr.	1
64.0 Gy (RBE)/8 fr.	4
66.0 Gy (RBE)/10 fr.	2
38.4 Gy (RBE)/12 fr.	1
40.0 Gy (RBE)/12 fr.	2
43.2 Gy (RBE)/12 fr.	2
44.0 Gy (RBE)/12 fr.	1
45.6 Gy (RBE)/12 fr.	8
48.0 Gy (RBE)/12 fr.	114
50.4 Gy (RBE)/12 fr.	7
51.6 Gy (RBE)/12 fr.	1
52.8 Gy (RBE)/12 fr.	101
55.2 Gy (RBE)/12 fr.	14
57.6 Gy (RBE)/12 fr.	14
64.8 Gy (RBE)/12 fr.	1
72.0 Gy (RBE)/12 fr.	1
52.0 Gy (RBE)/13 fr.	1
50.4 Gy (RBE)/14 fr.	1
52.8 Gy (RBE)/16 fr.	8
56.0 Gy (RBE)/16 fr.	1
57.6 Gy (RBE)/16 fr.	10
60.8 Gy (RBE)/16 fr.	1
64.0 Gy (RBE)/16 fr.	9
60.0 Gy (RBE)/20 fr.	1
65.0 Gy (RBE)/25 fr.	1
65.0 Gy (RBE)/26 fr.	6
70.2 Gy (RBE)/26 fr.	4
70.4 Gy (RBE)/32 fr.	2
74.0 Gy (RBE)/37 fr.	2

*RBE* relative biological effectiveness, *fr.* fractions

### Follow-up and evaluation

Following treatment, patients had follow-up visits 1 month after treatment, then every 3–4 months for the first 2 years, and every 3–6 months thereafter. Regular follow-up studies included physical examination and diagnostic imaging (i.e., CT and/or magnetic resonance imaging). Local control (LC) was defined as no evidence of LN regrowth within the irradiated field. Progression-free survival (PFS) was defined as the absence of locoregional or distant failure or death from any cause. Overall survival (OS) and adverse events after C-ion treatment were also evaluated. Late toxicity was graded according to the Common Terminology Criteria for Adverse Events (version 4.0) [18]. The highest toxicities occurring 3 months after commencing C-ion RT were defined as late toxicities in the present study.

### Statistical analysis

The incidences of LC, OS, and PFS were determined using the Kaplan–Meier method. These endpoints were calculated from the initiation of C-ion RT to the date of last follow-up or death from any cause. The log-rank test was used for univariate analyses. All factors with statistically significant associations in the univariate analysis were included in the multivariate analysis, and a Cox proportional hazards regression model was used. *p* values < 0.05 were considered significant; all statistical tests were two-sided. Statistical calculations were performed using the IBM SPSS Statistics 24 software (IBM, Armonk, NY, USA).

## Results

### Cohort characteristics

Patient and tumor characteristics are summarized in Table 2. The median patient age was 66 years (range, 29–90 years), and the median maximal LN diameter was 22 mm (range, 10–80 mm). The median disease-free interval (i.e., the time between primary treatment administration and the day of recurrence detection) was 16 months (range, 1–247 months). Sixty-six patients were diagnosed with squamous cell carcinoma (SCC), while the remaining 257 patients were diagnosed with non-SCC (including adenocarcinoma, adenosquamous carcinoma, and other carcinomas).

**Table 2:** Patient and tumor characteristics (*n* = 323)

Characteristic	*n* (%)
Age (years), median (range)	66 (29–90)
LN diameter in maximal length (mm), median (range)	22 (10–80)
Number of LNs	
1	215 (66.6)
2	65 (20.1)
3	15 (4.6)
≥ 4	28 (8.7)
History of radiotherapy to the lesion	
No (initial irradiation)	276 (85.4)
Yes (re-irradiation)	47 (14.6)
Disease-free interval (months), median (range)	16 (1–247)
Histology	
SCC	66 (20.4)
Non-SCC	257 (79.6)
Primary site of carcinomas	
Head and neck	17 (5.3)
Lung	99 (30.7)
Breast	4 (1.2)
Upper GI (esophagus, stomach, duodenum)	26 (8.0)
Lower GI (colon, rectum)	77 (23.8)
Pancreas	20 (6.2)
Liver, cholecyst, bile duct	11 (3.4)
Urinary system (kidney, renal pelvis, bladder)	8 (2.5)
Prostate	7 (2.2)
Uterine	38 (11.8)
Ovary	8 (2.5)
Primary unknown	1 (0.3)
Others (vagina, skin, chordoma, adrenal gland)	7 (2.2)

*LN* metastatic lymph node, *SCC* squamous cell carcinoma, *GI* gastrointestinal tract

### Treatment efficacy and prognostic factors

**Fig. 1:**
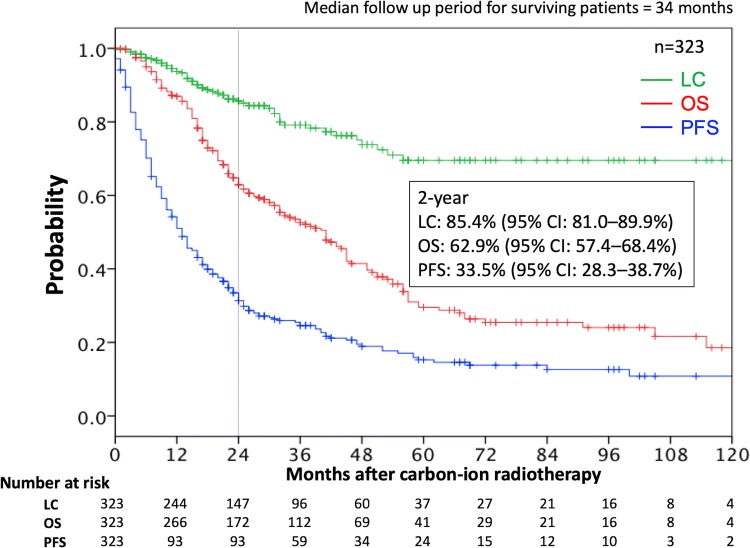
Kaplan–Meier curves of local control (green), overall survival (red), and progression-free survival (blue) for all 323 patients. The numbers at risk are shown below the figure. *CI* confidence interval, *LC* local control, *PFS* progression-free survival, *OS* overall survival

The median follow-up period was 34 months (range, 2–181 months) for surviving patients. The 2-year LC, OS, and PFS rates were 85.4% (95% confidence interval [CI] 81.0–89.9%), 62.9% (95% CI 57.4–68.4%), and 33.5% (95% CI 28.3–38.7%), respectively (Fig. 1). Site-specific clinical results (the 5 most common primary sites) are shown in Supplementary Table 1. Table 3 lists the results of the log-rank tests for prognostic factors. Regarding the setting of cutoff values in the univariate or multivariate analysis, median values were used as the cutoff for age (66 years) and disease-free interval (16 months). The cutoff value for LN diameter was set as 30 mm based on previous studies [19, 20]. In addition, the analyses for the number of LNs were conducted between 2 groups (1–3 versus ≥ 4) based on a previous study [21]. None of the analyzed factors, including LN diameter and histology, were significantly correlated with LC in the univariate analysis. However, the number of LN metastases was associated with PFS. Furthermore, the LN diameter (maximum length), number of LN metastases, and disease-free interval were associated with OS. The factors that were significant in the univariate analysis were then assessed to identify predictors of clinical outcomes using multivariate analyses based on the Cox proportional hazards model (Table 4). A smaller number (≤ 3) of LN metastases was associated with significantly better PFS (*p* = 0.047). Moreover, a smaller LN diameter (< 30 mm) (*p* = 0.011), smaller number (≤ 3) of LN metastases (*p* = 0.006), and longer disease-free interval (≥ 16 months) following primary therapy (*p* = 0.003) were associated with significantly better OS.

**Table 3:** Assessment of prognostic factors with univariate analysis

Factor	Number of patients	LC		PFS		OS	
		2-year (%)	*p* value	2-year (%)	*p* value	2-year (%)	*p* value
Age (years)			0.235		0.114		0.664
< 66	154	80.4		27.0		61.4	
≥ 66	169	89.6		35.3		64.2	
LN diameter in maximal length (mm)			0.485		0.056		0.010
< 30	229	85.4		34.2		68.8	
≥ 30	94	86.1		24.5		48.6	
Number of LNs			0.242		0.023		0.001
1–3	295	86.5		33.1		65.2	
≥ 4	28	67.3		12.5		38.9	
History of radiotherapy to the lesion			0.394		0.009		0.016
No (initial irradiation)	276	84.8		28.9		61.5	
Yes (re-irradiation)	47	88.9		45.7		70.7	
Histology			0.703		0.585		0.181
SCC	66	83.8		29.3		54.1	
Non-SCC	257	85.7		31.9		65.3	
Disease-free interval (months)			0.269		0.324		0.001
< 16	162	81.9		25.8		53.2	
≥ 16	161	88.6		37.0		72.6	

*LN* metastatic lymph node, *SCC* squamous cell carcinoma, *EQD*_*2*_ equivalent dose in 2-Gy fraction, *LC* local control, *PFS* progression-free survival, *OS* overall survival

**Table 4:** Assessment of prognostic factors with multivariate analysis

Factor	PFS		OS	
	*p* value	HR (95% CI)	*p* value	HR (95% CI)
LN size in maximal length < 30 mm			0.011	0.668 (0.490–0.912)
Number of LNs (1–3)	0.047	0.658 (0.435-0.995)	0.006	0.535 (0.341–0.837)
History of RT to the lesion (re-irradiation)	–		–	
Disease-free interval ≥ 16 months			0.003	0.629 (0.466–0.851)

*LN* metastatic lymph node, *SCC* squamous cell carcinoma, *PFS* progression-free survival, *OS* overall survival, *CI* confidence interval, *HR* hazard ratio

### Toxicities

All patients completed their planned C-ion RT except for 1 who developed grade 2 esophagitis; this patient received 50.4 Gy (RBE) in 14 fractions and ceased treatment. The esophagitis resolved after treatment, and no late toxicity was observed in this patient. As shown in Supplementary Table 2, 15 patients (4.6%) developed grade ≥ 2 late toxicities [Supplementary Table 2(A)]; of these, 1 developed severe (grade 3) late toxicity [Supplementary Table 2(B)]. The patient who developed grade 3 late toxicity had originally undergone surgery for tongue carcinoma; this patient received C-ion RT at a dose of 65.0 Gy (RBE) in 26 fractions for submandibular LN and subsequently developed grade 3 facial nerve disorder.

## Discussion

Ours is the first multicenter study to investigate the efficacy of C-ion RT in patients with LN oligo-recurrence. Few published data exist regarding the use of conventional RT in the context of LN oligo-recurrence. However, reports of the clinical outcomes of patients with LN oligo-recurrence who underwent SBRT have been published in recent years; Table 5 lists the published studies of patients with LN oligo-recurrence in which the median follow-up times were ≥ 12 months [19, 22–29]. Although these studies are heterogeneous in terms of site, primary histology, and dose, the control rates for treated metastatic LNs were 64–78%, except for patients with prostate cancer. The present study showed a 2-year LC of 85.4% despite the larger number of patients, the majority of whom had non-SCCs (79.6%) and among whom prostate cancer was rare (2.2%). Thus, C-ion RT can be a powerful focal therapy for patients with LN oligo-recurrence.

**Table 5:** Clinical outcomes of SBRT and present study for lymph node oligo-recurrence

Author (references)	Year	Number of patients	Primary disease	Dose fractionation	Median follow-up time (months)	Local control	Grade ≥ 3 late toxicity
Kim et al. [22]	2008	23	Colorectal	30–51 Gy/3 fr.	31	74% at 4 years	4% (Grade 4 in 1 patient)
Choi et al. [19]	2009	30	Uterine	33-45 Gy/3 fr.	15	67% at 4 years	20%
Kim et al. [23]	2009	7	Gastric	48 Gy/3 fr. (median)	26	NR	0%
Bignardi et al. [24]	2011	19	Mixed	45 Gy/6 fr. (median)	12	78% at 2 years	5%
Casamassima et al. [25]	2011	25	Prostate	30 Gy/3 fr. (most common)	29	90% at 3 years	0%
Bea et al. [26]	2012	41	Colorectal	48 Gy/3 fr. (median)	28	64% at 3 years	7%
Wang et al. [27]	2016	85	Mixed	45 Gy/5 fr. (most common)	27	77% at 5 years	7% (Grade 5 in 3 patients)
Napieralska et al. [28]	2016	18	Prostate	30 Gy/3 fr. (median)	16	70% at 2 years	NR
Jereczek-Fossa et al. [29]	2017	94	Prostate	33 Gy/3 fr. (median)	19	84% at 2 years	0%
Present study	2019	323	Mixed	48 Gy (RBE)/12 fr. (most common)	25 (34)^a^	85% at 2 years	0%^b^ (Grade 3 in 1 patient)

*SBRT* stereotactic body radiotherapy, *fr.* fractions, *RBE* relative biological effectiveness, *NR* not reported

^a^Median follow-up period for surviving patients

^b^0.31% (one out of 323 patients) for the record

Previous studies found that LN diameter and volume may be predictors of in-field control [19, 30]. The information in Table 5 implies that patients with LN oligo-recurrence of prostate cancer had better LC rates. Regarding the association between LC in patients with irradiated LNs and histological tumor type, Milano et al. reported a better LC rate in patients with breast cancer than in those with non-breast cancers [31]. However, other factors, such as LN diameter or histology, did not significantly affect LC in the univariate analysis in the present study. Notably, a history of RT at the site of recurrence did not compromise LC, which is consistent with recent studies investigating C-ion RT for LN recurrence [15, 16]. Additionally, our study revealed that C-ion RT for LN oligo-recurrence caused relatively few late toxicities, with only 1 patient developing grade 3 late toxicity. Thus, C-ion RT appears to be beneficial for conventionally difficult-to-cure patients with LN oligo-recurrence.

Several researchers reported an association between dose fractionation and in-field control when using SBRT for LN oligo-recurrence. Bae et al. found that a dose of 48 Gy or greater in 3 fractions was associated with better in-field control in a series of 41 patients with colorectal cancer metastases [26]. Conde-Moreno et al. proposed using dose fractionation schemes of either 6 fractions of 7.5 Gy or 3 fractions of ≥ 10 Gy based on criteria that depended on the tolerance of the surrounding structures [20]. However, univariate analysis revealed no significant difference in LC according to the C-ion RT dose in our study; this could be owing to the variety of maximum LN diameters, which ranged from 10 to 80 mm. Prospective trials to determine the optimal C-ion RT dose fractionation scheme for LN oligo-recurrence are warranted.

In our study, having ≤ 3 LN metastases was associated with significantly better PFS. Moreover, LNs with a diameter < 30 mm, having ≤ 3 LN metastases, and a disease-free interval ≥ 16 months after the initial therapy were associated with significantly better OS. These findings support the notion of an oligo-recurrence state in which aggressive local therapy can provide a survival benefit in a specific population. Regarding prognostic factors reported in the literature, Salama et al. reported that patients with ≤ 3 metastases had better PFS and OS than those with 4–5 metastases [21]. Although their cohorts included multisite extracranial oligo-recurrence that were not confined to LNs, our results support their findings. Jereczek-Fossa et al. reported that a larger LN volume compromised LC and predicted a poorer prognosis; an abdominal gross tumor volume of 17 cm^3^ or smaller predicted favorable PFS; and each additional 1 cm^3^ was associated with a 1% worsening in PFS [32]. Although LN diameter did not affect the LC rate in our study, it did affect OS. Thus, LN diameter may be a prognostic factor in certain patients with LN oligo-recurrence, as may the disease-free interval post-primary therapy. Zhang et al. reported that a disease-free interval of more than 12 months was significantly related to longer OS in patients with lung metastases who received SBRT [33]. Furthermore, other researchers have reported that a longer disease-free interval was significantly associated with longer OS in patients with extracranial oligo-recurrence or recurrent hepatocellular carcinoma following SBRT [34, 35]. These results, as well as those of our study, indicate that C-ion RT should be considered in patients with LN oligo-recurrence, particularly in patients with a longer disease-free interval.

The present study has some limitations. First, the study design was retrospective; therefore, various dose fractionation protocols were included. Second, the median follow-up period was short (25 months). Longer follow-up is needed to determine the long-term efficacy and toxicity of C-ion RT.

In conclusion, our study demonstrates that C-ion RT for LN oligo-recurrence appears to be effective and safe. In addition, C-ion RT may improve LC irrespective of tumor status and may produce a survival benefit in patients with LN oligo-recurrence. In particular, patients who have a small number of LN metastases (≤ 3), smaller LN diameter (< 30 mm), and longer disease-free interval (≥ 16 months) are most likely to achieve a survival benefit from C-ion RT.

## Electronic supplementary material

Below is the link to the electronic supplementary material.

**Supplementary material 1 (TIFF 34,610** **kb)** Representative treatment plan and tumor response to carbon-ion radiotherapy. Axial, coronal, and sagittal CT images with dose distribution are shown in (a), (b), and (c), respectively. GTV is highlighted in red. Yellow arrows indicate the direction of C-ion RT. (d): Fluorodeoxyglucose (^18^F) positron emission tomography-CT (FDG PET-CT) image before the start of treatment. (e): FDG PET-CT image at 6 months after treatment. Abbreviation: LN = lymph node

**Supplementary material 2 (DOCX 36** **kb)** Site-specific clinical results (the 5 most common primary sites)

**Supplementary material 3 (DOCX 38** **kb)** Late toxicities
