# Supplementary figures and images for: Carbon-ion radiotherapy for lymph node oligo-recurrence: a multi-institutional study by the Japan Carbon-Ion Radiation Oncology Study Group (J-CROS)
Source: Int J Clin Oncol. 2019 Apr 9;24(9):1143–50. doi: 10.1007/s10147-019-01440-y (PMC6687700; doi:10.1007/s10147-019-01440-y)

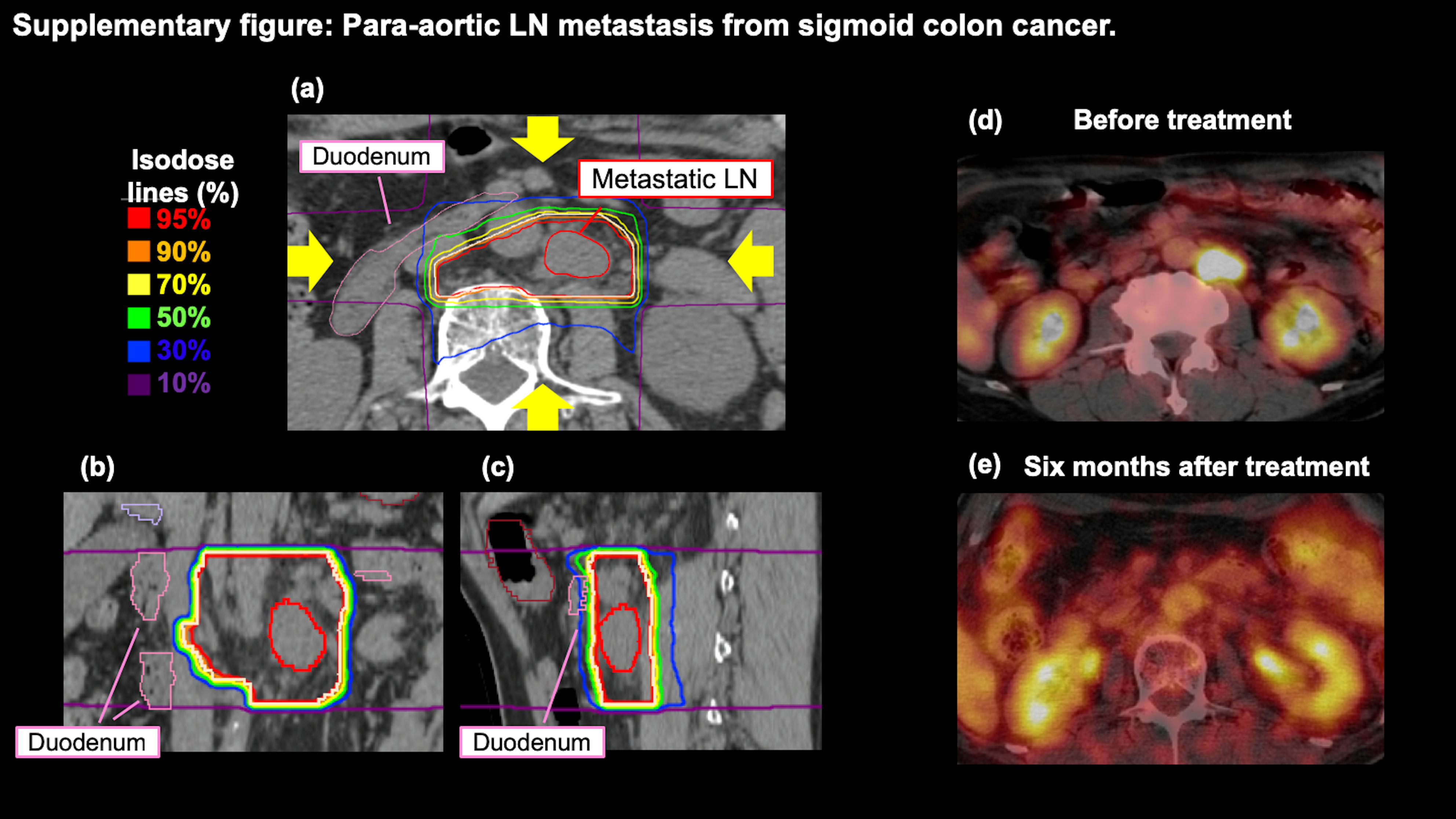

Supplement: Supplementary file 1 — Supplementary material 1 (TIFF 34,610 kb) Representative treatment plan and tumor response to carbon-ion radiotherapy. Axial, coronal, and sagittal CT images with dose distribution are shown in (a), (b), and (c), respectively. GTV is highlighted in red. Yellow arrows indicate the direction of C-ion RT. (d): Fluorodeoxyglucose (18F) positron emission tomography-CT (FDG PET-CT) image before the start of treatment. (e): FDG PET-CT image at 6 months after treatment. Abbreviation: LN = lymph node [file 10147_2019_1440_MOESM1_ESM.tiff]
