# Supplementary material for: Carbon-ion radiotherapy for lymph node oligo-recurrence: a multi-institutional study by the Japan Carbon-Ion Radiation Oncology Study Group (J-CROS)
Source: Int J Clin Oncol. 2019 Apr 9;24(9):1143–50. doi: 10.1007/s10147-019-01440-y (PMC6687700; doi:10.1007/s10147-019-01440-y)
Supplement: Supplementary file 2 — Supplementary material 2 (DOCX 36 kb) Site-specific clinical results (the 5 most common primary sites) [file 10147_2019_1440_MOESM2_ESM.docx]

**Supplementary Table 1. Site-specific clinical results (the 5 most common primary sites).**

| Primary site of carcinomas | Number of patients | LC |  | PFS |  | OS |
| --- | --- | --- | --- | --- | --- | --- |
|  |  | **2-year (%)** |  | **2-year (%)** |  | **2-year (%)** |
| Lung | 99 | 93.2 |  | 36.1 |  | 59.3 |
| Lower GI (colon, rectum) | 77 | 80.6 |  | 27.1 |  | 73.6 |
| Uterus | 38 | 87.7 |  | 41.9 |  | 74.5 |
| Upper GI (esophagus, stomach, duodenum) | 26 | 81.9 |  | 26.9 |  | 53.6 |
| Pancreas | 20 | 66.5 |  | 6.3 |  | 19.4 |

Abbreviations: LC = local control; PFS = progression-free survival; OS = overall survival; GI = gastrointestinal tract.
