# Supplementary material for: Carbon-ion radiotherapy for lymph node oligo-recurrence: a multi-institutional study by the Japan Carbon-Ion Radiation Oncology Study Group (J-CROS)
Source: Int J Clin Oncol. 2019 Apr 9;24(9):1143–50. doi: 10.1007/s10147-019-01440-y (PMC6687700; doi:10.1007/s10147-019-01440-y)
Supplement: Supplementary file 3 — Supplementary material 3 (DOCX 38 kb) Late toxicities [file 10147_2019_1440_MOESM3_ESM.docx]

**Supplementary Table 2. Late toxicities**

(A) Summary of late toxicities.

| Subgroup | Grades 0–1 | Grade 2 | Grade 3 | ≥Grade 4 |
| --- | --- | --- | --- | --- |
| Initial irradiation (n = 276) | 265 | 10 | 1 | 0 |
| Re-irradiation (n = 47) | 43 | 4 | 0 | 0 |

(B) List of patients who developed Grade 2 or higher toxicity.

| **No.** | **Primary disease** | **Treatment site** | **Dose/fractions** | **LN diameter (mm)** | **Grade** | **Detail** |
| --- | --- | --- | --- | --- | --- | --- |
| *Initial irradiation* | | | | | | |
| 1 | Lung ca. | Mediastinum LN | 43.2 Gy (RBE)/12 fr. | 29 | 2 | Pericardial effusion |
| 2 | Lung ca. | Hilar LN | 48.0 Gy (RBE)/12 fr. | 22 | 2 | Lung atelectasis |
| 3 | Rectal ca. | Abdominal LN | 48.0 Gy (RBE)/12 fr. | 35 | 2 | Duodenitis |
| 4 | Lung ca. | Mediastinum LN | 48.0 Gy (RBE)/12 fr. | 18 | 2 | Rib fracture |
| 5 | Rectal ca. | Abdominal LN | 52.8 Gy (RBE)/12 fr. | 10 | 2 | Duodenitis |
| 6 | Uterus ca. | Abdominal LN | 52.8 Gy (RBE)/12 fr. | 14 | 2 | Lumber fracture |
| 7 | Lung ca. | Mediastinum LN | 52.8 Gy (RBE)/12 fr. | 22 | 2 | Esophagitis |
| 8 | Lung ca. | Mediastinum LN | 52.8 Gy (RBE)/12 fr. | 17 | 2 | Thrombosis in subclavian vein |
| 9 | Endometrioid ca. | Pelvic LN | 52.8 Gy (RBE)/12 fr. | 30 | 2 | Right leg edema |
| 10 | Esophageal ca. | Cervical LN | 57.6 Gy (RBE)/16 fr. | 66 | 2 | Skin atrophy |
| 11 | Tongue ca. | Cervical LN | 65.0 Gy (RBE)/26 fr. | 40 | 3 | Peripheral motor neuropathy |
| *Re-irradiation* | | | | | | |
| 1 | Uterus cervical ca. | Pelvic LN | 48.0 Gy (RBE)/12 fr. | 15 | 2 | Enterocolitis |
| 2 | Prostate ca. | Pelvic LN | 51.6 Gy (RBE)/12 fr. | 16 | 2 | Peripheral sensory neuropathy |
| 3 | Breast ca. | Supraclavicular LN | 57.6 Gy (RBE)/12 fr. | 40 | 2 | Peripheral sensory neuropathy |
| 4 | Rectal ca. | Hilar LN | 72.0 Gy (RBE)/12 fr. | 56 | 2 | Lung atelectasis |

**Supplementary Table (cont). Late toxicities**

Abbreviations: fr. = fractions; LN = metastatic lymph node; ca. = carcinoma/cancer; RBE = relative biological effectiveness.
